# Disruption of Myc-Max Heterodimerization with Improved Cell-Penetrating Analogs of the Small Molecule 10074-G5

**DOI:** 10.18632/oncotarget.1108

**Published:** 2013-06-22

**Authors:** Huabo Wang, Jay Chauhan, Angela Hu, Kelsey Pendleton, Jeremy L. Yap, Philip E. Sabato, Jace W. Jones, Mariarita Perri, Jianshi Yu, Erika Cione, Maureen A. Kane, Steven Fletcher, Edward V. Prochownik

**Affiliations:** ^1^ Section of Hematology/Oncology, Children's Hospital of Pittsburgh of UPMC; ^2^ Department of Pharmaceutical Sciences, University of Maryland School of Pharmacy, Baltimore, MD, USA; ^3^ PharmD Program, University of Maryland School of Pharmacy, Baltimore, MD, USA; ^4^ Department of Pharmaco-Biology, University of Calabria, Rende (CS), Italy; ^5^ University of Maryland Marlene and Stewart Greenebaum Cancer Center, Baltimore, MD, USA; ^6^ Department of Microbiology and Molecular Genetics, The University of Pittsburgh School of Medicine; ^7^ The University of Pittsburgh Cancer Institute, Pittsburgh, PA

**Keywords:** 10058-F4, 10074-G5, intrinsically disordered proteins, JQ-1, BRD4

## Abstract

The c-Myc (Myc) oncoprotein is a high-value therapeutic target given that it is deregulated in multiple types of cancer. However, potent small molecule inhibitors of Myc have been difficult to identify, particularly those whose mechanism relies on blocking the association between Myc and its obligate heterodimerization partner, Max. We have recently reported a structure-activity relationship study of one such small molecule, 10074-G5, and generated an analog, JY-3-094, with significantly improved ability to prevent or disrupt the association between recombinant Myc and Max proteins. However, JY-3094 penetrates cells poorly. Here, we show that esterification of a critical *para*-carboxylic acid function of JY-3-094 by various blocking groups significantly improves cellular uptake although it impairs the ability to disrupt Myc-Max association *in vitro*. These pro-drugs are highly concentrated within cells where JY-3-094 is then generated by the action of esterases. However, the pro-drugs are also variably susceptible to extracellular esterases, which can deplete extracellular reservoirs. Furthermore, while JY-3-094 is retained by cells for long periods of time, much of it is compartmentalized within the cytoplasm in a form that appears to be less available to interact with Myc. Our results suggest that persistently high extracellular levels of pro-drug, without excessive susceptibility to extracellular esterases, are critical to establishing and maintaining intracellular levels of JY-3-094 that are sufficient to provide for long-term inhibition of Myc-Max association. Analogs of JY-3-094 appear to represent promising small molecule Myc inhibitors that warrant further optimization.

## INTRODUCTION

Although small molecule inhibitors of protein-protein interactions (PPIs) are of significant interest for the therapy of cancer and other diseases, their identification and clinical implementation have remained elusive for several reasons. First, unlike the active sites of enzymes, which typically accommodate inhibitors with high affinity and specificity, the interacting surfaces of proteins to which most PPI inhibitors are targeted are typically large and featureless [1-5]. Moreover, the disruption or prevention of PPIs with small molecules requires that the latter be able to overcome the high free energy of association between the protein partners [2, 6, 7]. Despite these challenges, some success has been obtained with small molecules such as the nutlins, which disrupt the interaction between the TP53 tumor suppressor and its negative regulator HDM2 [8, 9] as well as ABT-737 and various synthetic α-helix mimetics, which abrogate the interaction between pro- and anti-apoptotic Bcl-2 family members and accelerate programmed cell death [10, 11].

One prominent target of potentially high therapeutic value in cancer is the c-Myc (Myc) oncoprotein, a bHLH-ZIP transcription factor that is over-expressed and/or deregulated by many different tumor types [12-14]. Numerous studies have shown Myc to be necessary for the sustained proliferation and/or survival of various transformed cell types and that its inhibition promotes tumor regression *in vivo*, even when the inciting oncogenic stimulus is not typically associated with Myc de-regulation [15-19]. That even long-term Myc silencing *in vivo* is associated with surprisingly mild and generally reversible side effects [19] further enhances the appeal of direct Myc inhibition as a rational therapeutic option. Such findings temper the concern that the pharmacologic targeting of Myc, which is seldom mutated in cancer and is expressed by virtually all proliferating cells, would lead to unacceptable systemic toxicities[6].

We and others have identified small molecules that perturb the interaction between Myc and its obligate bHLH-ZIP heterodimerization partner Max. This leads to a loss of sequence-specific DNA binding, transcriptional regulation and various Myc-dependent phenotypes [6, 7, 20-28]. We have further demonstrated that some of these so-called “Myc inhibitors” bind to short, intrinsically disordered (ID) segments [29-31] within the bHLH-ZIP domain of the unstructured Myc monomer [32-34]. Ligand binding induces localized conformational changes that inhibit subsequent heterodimerization with Max or, in some cases, drive the ordered helical structure of the Myc-Max ZIP domain into a more disordered state [33-35]. At higher concentrations, Myc inhibitors also disrupt pre-existing Myc-Max heterodimers both free and in association with consensus E-box-containing double-stranded oligonucleotides [21, 23, 33]. Biophysical techniques including fluorescence polarization, circular dichroism, and NMR spectroscopy have provided direct evidence for at least 3 Myc inhibitor binding sites on the bHLH-ZIP domain with binding at each ID site occurring independently of the occupancy status of the others [33, 34].

Whereas the Myc inhibitors we originally identified tend to be quite specific, their *in vitro* affinities for Myc are relatively low and their IC_50_s for Myc-over-expressing cells are high [21, 23]. Moreover, in the two cases where pharmacologic properties have been studied, rapid *in vivo* metabolism and poor tumor penetration likely explain the lack of significant therapeutic benefit [36, 37]. This has led to the development of analogs with improved pharmacologic profiles, more potent Myc-binding and greater specificity. For example, 10058-F4, one of the originally reported parental Myc inhibitors [21], binds to a ca. 10 amino acid ID segment of Myc spanning the bHLH and ZIP junction (residues 402-412) [33, 34]. Nearly one-third of a large number of 10058-F4 analogs retained Myc-binding activity, with several being significantly more active than the parental compound. Improved anti-proliferative activity of some of these against Myc-over-expressing tumor cells generally correlated with a reduction in intracellular Myc-Max heterodimers [23]. A subsequent search employing a 3-D pharmacophore model identified additional structurally diverse Myc inhibitors some of which also showed improved binding to Myc and greater potency against tumor cells compared to 10058-F4 [25]. The ease with which active 10058-F4 analogs were identified in these studies was explained by subsequent work demonstrating that the proposed models of Myc inhibitors bound to their target sites [33] actually represent the average of an ensemble of dynamic structures, each with similarly low free energies of binding, that occur as a result of the plasticity of the peptide binding site as previously proposed for ID regions [29-31].

Another of our originally described Myc inhibitors, 10074-G5 (N-([1,1’-biphenyl]-2-yl)-7-nitrobenzo[c][1,2,5]oxadiazol-4-amine) [21] binds to a more N-terminal region of Myc’s HLH domain ~35 residues removed from the 10058-F4 binding site [34]. Because the reported model of Myc-bound 10074-G5 is also based on the average of multiple dynamic structures of an ID domain [34], we predicted that, as in the case of 10058-F4, more potent analogs of 10074-G5 should be attainable. To this end we have recently reported a structure-activity relationship study of 10074-G5 [28] that identified a new analog, dubbed JY-3-094, with a nearly 5-fold improvement in its ability to perturb the heterodimerization of Myc-Max recombinant proteins. In testimony to its specificity, JY-3-094 had no effect on Max homodimers [28]. However, it appeared to penetrate cells poorly, with high growth inhibitory IC_50_s against cell lines such as HL60 promyelocytic leukemia and Daudi Burkitt lymphoma, which express high Myc levels and tend to be quite sensitive to other Myc inhibitors [23, 28].

In the current study, we utilized JY-3-094 as a starting point to investigate how to modify its structure so as to optimize its cellular uptake and distribution, its disruption of Myc-Max heterodimers and its anti-proliferative effect. We found that esterification of a critical *para*-carboxylic acid function of JY-3-094 significantly improved cellular uptake. While these alterations tended to inhibit Myc-Max disrupting activity *in vitro*, the compounds were converted to JY-3-094 following their cellular uptake, although this occurred at highly variable rates. Live cell confocal imaging and mass spectroscopic analysis of the intracellular fate of these pro-drugs suggested that their ability to inhibit Myc-Max heterodimerization and proliferation was collectively dependent on the extent to which they could be converted to JY-3-094, their intracellular retention and their accessibility to Myc. Together, these studies show that directed chemical modifications designed to optimizing cellular uptake and retention represent a useful strategy for maximizing intracellular levels of Myc inhibitors.

## RESULTS AND DISCUSSION

### 10074-G5 is highly sensitive to structural alterations

**Figure 1:**
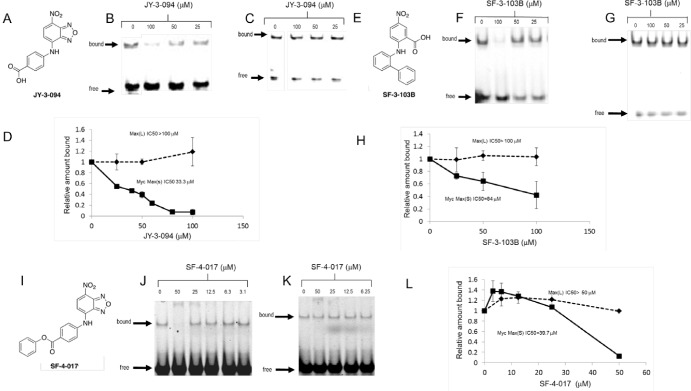
A-D, Properties of JY-3-094 A, Structure of the 10074-G5 analog JY-3-094. B, Representative EMSA performed with Myc-Max(S) heterodimers [23,28]. C, An identical EMSA performed with Max(L) homodimers. D, Quantification of EMSA results. Triplicate gels from B and C were scanned as previously described [23]. The mean values obtained at each compound concentration ± 1 S.E. for both Myc-Max(S) (panel B) and Max(L) EMSAs (panel C) are graphed. **E-H, Properties of SF-3-103B**. E, Structure of SF-3-103B. F, Representative EMSA results performed with Myc-Max(S) heterodimers. G, Representative EMSA performed with Max(L) homodimers. H, Quantification of EMSAs. Triplicate gels were scanned and binding was quantified as described for D. The mean values obtained at each compound concentration ± 1 S.E. for both Myc-Max(S) and Max(L) EMSAs are graphed. **I-L, Properties of SF-4-017.** I, Structure of SF-4-017. J, Representative EMSA results performed with Myc-Max(S) heterodimers. K, Representative EMSA performed with Max(L) homodimers. L, Quantification of EMSAs performed as described for D and H.

Unlike the case of analogs of the Myc inhibitor 10058-F4 [21], a substantial fraction of which retain the ability to disrupt Myc-Max heterodimers [23], only 2 of 24 tested 10074-G5 analogs, which we refer to as “Group A” compounds, did so (Supplementary Fig. S1 and ref. 28). IC_50_s for these compounds (JY-3-094 and SF-3-103B), determined in Myc-Max(S) EMSAs were 4.4- and 1.7-fold lower, respectively, than for 10074-G5 (Fig. 1 A–H and Supplementary Fig. S5). The specificity of JY-3-094 and SF-3-103B for Myc-Max(S) heterodimers was also demonstrated by showing that neither compound appreciably affected DNA binding by Max(L) homodimers (IC_50_s >100 μM) whose free energy of association is actually less than that of Myc-Max heterodimers [38]. Despite these improvements over the parental 10074-G5 compound neither analog showed appreciable activity against HL60 promyelocytic leukemia or Daudi Burkitt lymphoma cells in a standard proliferation assay (IC_50_s >50 μM, Table 1) [21, 23]. These results indicated that the requirements for 10074-G5 binding to Myc are more stringent than those for 10058-F4 and that further modification was needed to optimize cell-based activities. We reasoned that the ionizable carboxylic acid groups of JY-3-094 and SF-3-103B might be responsible for impeding cell entry.

**Table 1:** IC_50_s of JY-3-094 and Group B Myc Inhibitors

Compound	IC_50_ (μM)		HL60	Daudi
JY-3-094	>100	>100
3JC-91-1	20	7.8
3JC-91-2	7.2	3.5
3JC-91-3	>25	7.4
3JC-91-5	31.9	30.7
3JC-91-7	8.5	1.9
SF-4-017	9.6	3.1

Each of the indicated compounds was tested against logarithmically growing HL60 and Daudi cells in a standard 10-point serial dilution assay with each point being tested in quadruplicate in a standard MTT-based assay [23,28].

### Improved activities of esterified pro-drugs based on JY-3-094

In an effort to enhance the biological activity of JY-3-094, we esterified its *para-*carboxylic acid moiety with various blocking groups designed to facilitate cellular uptake while allowing hydrolysis by intracellular esterases back to JY-3-094 (Group B compounds-Supplementary Fig. S2). As expected, each of these modified compounds inhibited HL60 and/or Daudi cell proliferation (Table 1) to variable degrees although only one, SF-4-017, also retained activity in EMSAs (Fig. 1I-L). Based on the extreme sensitivity of 10074-G5 to structural changes (Supplementary Fig. S1), it was not surprising that most Group B compounds also failed to retain this latter property. Together, these findings suggested that esterification of the carboxylic acid group, while generally preventing the compound from disrupting Myc-Max *in vitro*, facilitates its cellular uptake whereupon intracellular esterase-mediated cleavage of the blocking group regenerates JY-3-094 with its specific Myc-Max disrupting activity.

**Figure 2:**
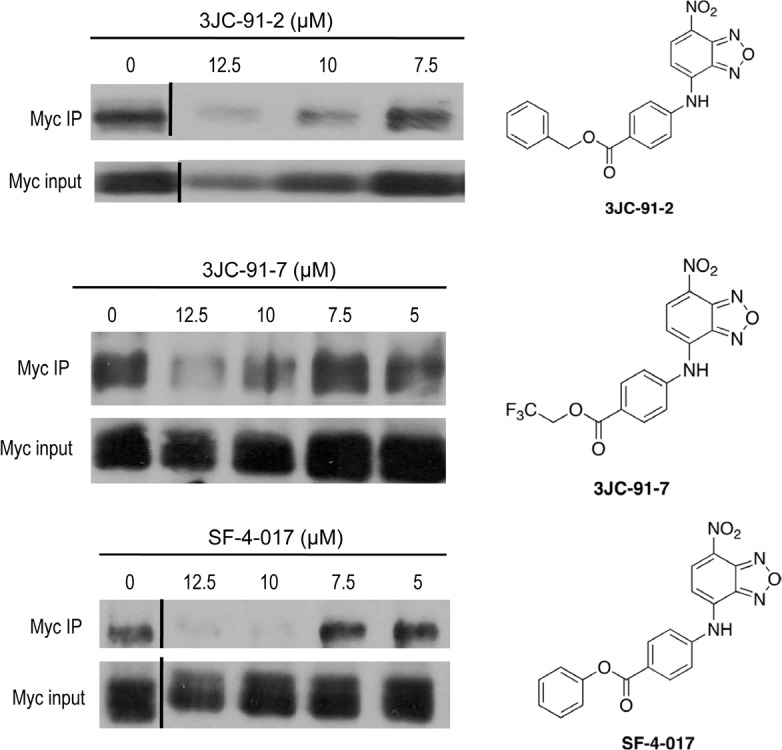
Co-IPs of Myc-Max complexes from HL60 cells Logarithmically growing HL60 cells were treated for 4-6 hr with the indicated concentrations of 3JC-91-2, 3JC-91-7 and SF-4-017. Total cell lysates were then incubated with an anti-Max antibody as previously described [23] and precipitated with protein-G-agarose. Precipitates were boiled, resolved by SDS-PAGE, transferred to PVDF membranes and probed with an anti-Myc monoclonal antibody (Myc IP). Blots were developed using an enhanced chemiluminesce protocol. The lower panel of each blot shows the total input of Myc protein prior to co-IP. Chemical structures of each compound are shown adjacent to their respective blots.

To examine this more carefully, we next asked if we could demonstrate intracellular disruption of Myc-Max heterodimers with select Group B compounds. We therefore exposed HL60 cells to three of the compounds, precipitated total cell lysates with an anti-Max antibody and performed western blotting to identify co-immunoprecipitated Myc protein[23]. In each case, we observed reproducible, dose-dependent inhibition of *in situ* Myc-Max heterodimer formation at compound concentrations that correlated well with growth inhibition assays (Fig. 2 and Table 1). These findings are consistent with the idea that, while Group B compounds are generally poor Myc-Max disruptors in EMSAs, they reacquire this activity following intracellular hydrolysis to JY-3-094.

### Live cell uptake of 10074-G5 and its analogs

**Figure 3:**
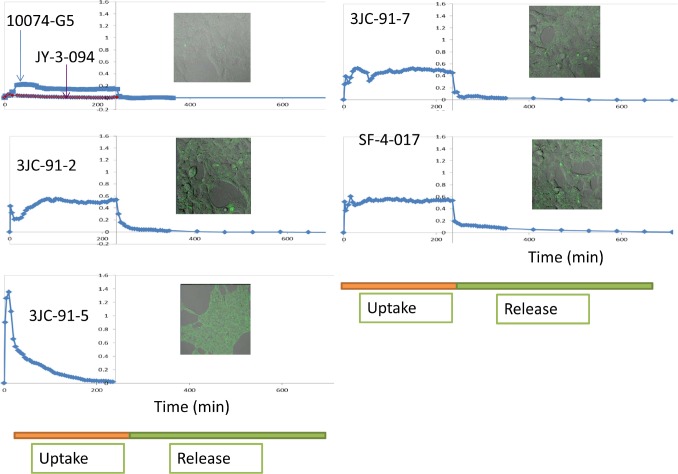
Live cell uptake and release of 10074-G5 and select Group B compounds All experiments were performed on semi-confluent monolayer cultures of HEK cells plated the day before. Myc inhibitors were added to cultures at final concentrations of 10 μM at 37C with quantification of uptake commencing immediately afterwards. Live cell images were obtained at least every 5 min. At the end of the uptake period (200 min), monolayers were washed free of compounds and the incubation was then continued in compound-free medium for the remainder of the study. Inserts show typical images of cells taken upon reaching peak compound levels.

We next took advantage of the fact that 10074-G5, JY-3-094 and all Group B analogs are fluorescent (Supplementary Fig. S6). This permitted a real-time comparison of their cellular uptake and distribution as well as their release and/or degradation to non-fluorescent metabolites following their removal. For these studies, we used live cell confocal microscopy of adherent human embryonal kidney (HEK) cells. As seen in Fig. 3 and Supplementary video 1, an extremely low level of 10074-G5 uptake was observed whereas the apparent uptake of JY-3-094 was below the limits of detection. In contrast, 3 of the 4 tested Group B compounds were taken up rapidly, persisted within cells at high levels and were then quickly released and/or converted to non-fluorescent metabolites following compound removal. Consistent with them having been optimized for cellular uptake, these compounds showed 2-3-fold greater fluorescence intensities upon reaching equilibrium relative to 10074-G5 and immeasurably better uptake relative to JY-3-094. The exception to this behavior occurred with 3JC-91-5. Although this compound was taken up as rapidly as the others, and even attained ~7-fold higher peak intracellular levels, its fluorescent signal rapidly dissipated during the remainder of the uptake phase. This behavior suggested that 3JC-91-5 was being converted to a non-florescent compound and/or to JY-3-094 within cells, which, upon release, would not be subject to re-uptake. To confirm that the loss of 3JC-91-5 fluorescence during the uptake phase of the above experiments was not attributable to a change in cellular behavior, the spent medium from cells treated with this compound was removed after 4 hr. and placed onto fresh cells. As seen in Supplementary Fig. S7A, these cells failed to show any evidence for compound uptake. In contrast, the same experiment performed with 3JC-91-2, showed that the spent medium contained sufficient residual compound such that its uptake by fresh recipient cells could again be detected (Supplementary Fig. S7B). These results suggest that 3JC-91-5 was being rapidly metabolized. To investigate precisely where this was occurring, we repeated the above uptake study with 3JC-91-5 in medium lacking serum. We observed the same overall fluorescence pattern previously seen in the presence of serum, namely the attainment of an initially high peak level of intracellular fluorescence followed by a decline in signal, albeit at a slower rate than seen previously (Supplementary Fig. S7C). This suggested that the more rapid decline of fluorescence in serum-containing medium was due to the presence of serum esterases. To address this directly, 3JC-91-5 was incubated in serum-containing medium for 6 hr in the absence of cells. We then utilized MS to determine whether any of the compound had been hydrolyzed to JY-3-094. As seen in Supplementary Fig. S7D, >80% of 3JC-91-5 was hydrolyzed to JY-3-094. Together with the cell-based assays, these results suggest that 3JC-91-5 is hydrolyzed to JY-3-094 both intra- and extra-cellularly. The extreme rapidity of the latter process, coupled with the apparent poor uptake of JY-3-094 appears sufficient to explain the rapid loss of the fluorescent signal following the addition of 3JC-91-5 to cells (Fig. 3). That 3JC-91-5 also initially attains higher intracellular concentrations than other Group B compounds also likely contributes to its faster intracellular hydrolysis.

Interestingly, all compounds that could be examined by live cell confocal microscopy localized primarily to the cytoplasm where they distributed both diffusely and in a more concentrated or “speckled” pattern (Fig. 3 inserts). This suggested that the primary site of interaction of 10074-G5 and Group B compounds with Myc is in the former compartment where they associate with the oncoprotein prior to its nuclear translocation. This does not rule out the possibility that nuclear concentrations of compounds, while presumably less than those attained in the cytoplasm, are nonetheless also sufficiently high to disrupt Myc-Max heterodimers in that compartment as well.

### Further mass spectroscopic evaluation of Group B compounds

**Figure 4:**
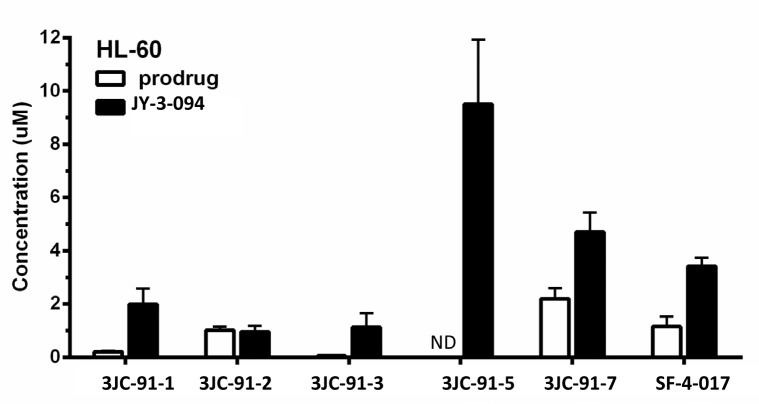
LC-MS/MS quantification of intracellular prodrug and metabolite in HL-60 cells Cells were cultured in the presence of 10 μM prodrug for 72 h. Intracellular prodrug and metabolite (JY-3-094) levels were then quantified by LC-MS/MS. All data are expressed as mean ± standard deviation with N=3 for each prodrug treatment. ND = not detectable.

Based on the foregoing findings, we asked if we could simultaneously detect intracellular Group B prodrugs and JY-3-094, the common product of esterase-mediated hydrolysis. HL-60 cells were therefore cultured in the presence of 10 μM of each prodrug for 72 h; its intracellular concentrations and that of JY-3-094 were then quantified by LC-MS/MS (Fig. 4). Since our aim was to evaluate the extent of ester hydrolysis of each prodrug to active JY-3-094, we did not search for other metabolites. Given that different prodrugs may be internalized and cleared at different rates, this approach also did not afford the opportunity to correlate total amounts of prodrug with total uptake. Exposure to 10 μM of the acetoxymethyl ester 3JC-91-5 afforded 9.5 μM intracellular concentration of metabolite (JY-3-094) with no detectable 3JC-91-5, suggesting 95% uptake of the prodrug, its complete conversion to JY-3-094 and its long-term intracellular retention in unmodified form. Similarly high rates of conversion were seen with methyl and ethyl esters (3JC-91-1 and 3JC-91-3, respectively), followed closely by phenol ester SF-4-017 and then trifluoroethyl ester 3JC-91-7. Similar degrees of conversion were observed when the above studies were repeated in MDA-MB-231 breast cancer cells (Supplementary Fig. S8).

The rate of metabolism of an ester prodrug depends on the structures of both the acid and the alcohol component. A simple methyl ester may be metabolized poorly if the acid portion is not well-accommodated in the active sites of esterases. The virtually complete conversion of 3JC-91-5 to JY-3-094 is consistent with the former compound being a metabolically-active acetoxymethyl ester as this side chain tends to be efficiently hydrolyzed through a cascade reaction in which metabolism of the terminal and sterically accessible acetyl group by esterases is pursued by a spontaneous chemical reaction to liberate formaldehyde and the drug [39]. Together with its poor activity in EMSAs, this suggested that the cytotoxicity of 3JC-91-5 as well as most other Group B members was largely attributable to their intracellular conversion to JY-3-094. On the other hand, given that SF-4-017 is also modestly active in EMSA-based assays (Fig. 1J), its cellular effects may be explained by a combination of the prodrug and JY-3-094.

The finding that high intracellular concentrations of JY-3-094 could be detected by MS following a 3 d exposure to 3JC-91-5 initially appeared to conflict with our prior observation that intracellular fluorescence of 3JC-91-5 persisted for only 2-3 hr following its addition to cells (Fig. 3). In order to resolve this discrepancy, we repeated the emission profiles of JY-3-094 in aqueous medium rather than DMSO. As seen in Supplementary Fig. S9 JY-3-094 lost virtually all its fluorescence in PBS whereas compounds 3JC-91-2 and SF-4-017, included as controls, retained this property despite some change in the appearance of the fluorescence profile. Taken together with our results obtained by MS, these findings indicate that the rapid loss of fluorescence signals seen following the removal of Group B compounds from cells (Fig. 3) is likely due to the release of non-hydrolyzed intracellular compound as well as to the lack of fluorescent signal of JY-094 in an aqueous environment at physiologic pH following its hydrolytic conversion. We believe this differential fluorescent behavior of JY-3-094 results from the ionization of its carboxylic acid group (pK_a_~5) in PBS in a manner analogous to that which occurs with compounds such as phenol and tyrosine upon ionization of their respective hydroxyl groups at high pH [40].

### A model for Myc inhibitor action

**Figure 5:**
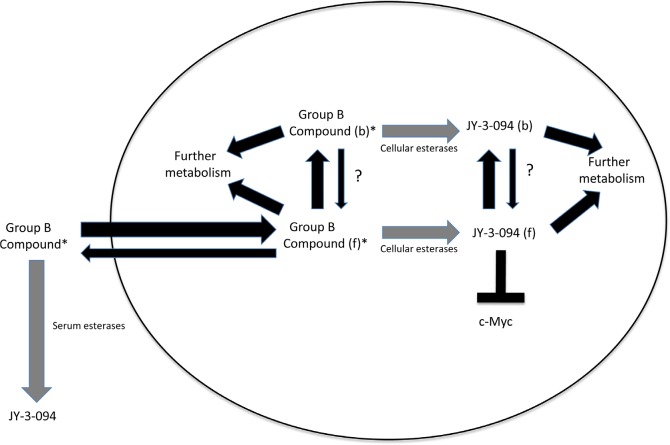
Model for Group B compound activity Esterification of JY-3-094 to yield Group B compounds greatly promotes cellular uptake but eliminates Myc inhibitory activity. Group B compounds are preferentially concentrated in cells but are susceptible to esterases (grey arrows) at rates that are determined by the identity of the ester group and the amounts and specificities of the esterases. Group B compounds are also susceptible to extracellular serum esterases, with highly esterase-susceptible compounds such as 3JC-91-5 being particularly prone to conversion to JY-3-094 (Fig. 3 and Suppl. Fig. S7). Upon entering cells, Group B compounds may either remain free within the cytoplasm (f) or become localized in a speckled pattern (Fig. 3) that presumably reflects their binding to proteins (b). The rates at which ester hydrolysis of free and bound compounds occur need not be identical although they are depicted as being so here. The hydrolyzed product, JY-3-094, remains bound and unable to interact with Myc (thin arrows). It is not currently known how freely interchangeable the free and bound states are (?). That virtually all of a compound such as 91-5 is converted intracellularly to JY-3-094 yet is a relatively weaker inhibitor of proliferation (Table 1) supports the idea that the bound form of JY-3-094 remains unavailable for Myc interaction despite its prolonged intracellular half-life. In contrast, the more efficient inhibition of Myc-Max heterodimers and proliferation by compounds such as 3JC-91-1, 3JC-91-2 and 3JC-91-3, despite the lower total levels of JY-3-094 suggests that higher levels of the free form of the latter compound are maintained by virtue of the persistence of an extracellular reservoir of the former compounds. (*) indicates that the compound is fluorescent under aqueous conditions.

The results presented here are most consistent with the model depicted in Fig. 5 in which Group B compounds are taken up and concentrated within cells or hydrolyzed extracellularly to JY-3-094. Whereas the former process occurs at similar initial rates in all cases (Fig. 3), hydrolytic rates differ and are dictated by the identity of the ester linkage. When hydrolytic rates are low, sufficient extracellular Group B compound persists so as to establish a longer-term equilibrium between freely interchangeable intra- and extracellular pools. Alternatively, when ester hydrolysis is rapid, as in the case of 3JC-91-5, the extracellular pool is rapidly depleted and reduced to a form (JY-3-094) that is both poorly taken up and rendered incapable of being visualized in its aqueous environment. Group B compounds are similarly susceptible to intracellular esterases, thus further contributing to the loss of signal. Group B compounds and JY-3-094 appear to partition between a diffuse or “free” cytoplasmic state and a more concentrated or “speckled” state, with Group B compounds appearing equally prone to hydrolysis in either state (Fig. 3 and Supplementary video 1). It seems reasonable to speculate that the diffusely distributed JY-3-094 represents the form that is the most available to inhibit Myc. The degree to which this is achieved likely reflects a complex combination of factors that includes the extent of Group B compound uptake, its level and length of persistence in the extracellular compartment, its rate of both extra- and intra-cellular hydrolysis to JY-3-094, its subcellular distribution and the extent to which JY-3-094 can effectively interact with Myc. Another factor, not examined here, includes the rate at which Group B compounds are differentially metabolized within cells to compounds other than JY-3-094.

## SUMMARY

The current study demonstrates that substantial lead optimization of 10074-G5 can be achieved by a combination of pharmacophore identification, which yielded JY-3-094 [28] and cellular uptake optimization, which yielded Group B members. By blocking the *para*-carboxylic group of JY-3-094 and thus neutralizing a net negative charge that likely contributes to poor cellular uptake, we greatly enhanced the activity of this analog while sacrificing its ability to disrupt Myc-Max heterodimers until being hydrolyzed back to JY-3-094. Together, these approaches improved compound potency by almost 5-fold when measured by EMSA and by as much as 30-50-fold when measured by cell proliferation assays (Table 1). Despite the difficulty of maintaining activity in these assays following any modification of the JY-3-094 core structure, its esterified analogs were actually considerably more potent when compared to a larger set of Myc inhibitors derived from 10058-F4, which binds to a different site on the Myc bHLH-ZIP domain [21, 23, 25]. Our present results are particularly noteworthy given that 10058-F4 contains a pan-assay interference rhodanine moiety and an α,β unsaturated ketone group that could function as a Michael acceptor [41, 42]. JY-3-094 and its ester pro-drugs suffer from neither of these potential drawbacks, thus suggesting that they may be better candidates for more advanced pharmaceutical development.

The rapidity with which 3JC-91-5 was hydrolyzed to JY-3-094 underscores the importance of selecting the most appropriate blocking group for the purpose of esterification. Excessive hydrolysis, particularly by extracellular esterases, can deplete the reservoir of pro-drug by converting it to a form that restricts cellular entry. Future attempts at compound optimization should perhaps also focus on the generation of prodrugs that are better retained following their intracellular hydrolysis. Compounds with a more diffuse cytoplasmic distribution might also afford better accessibility to Myc, thus raising their effective intracellular concentrations.

Our combined results suggest that the ability of any given Group B compound to inhibit cellular proliferation via the disruption of Myc-Max association is the result of several factors all working in concert, only some of which have been quantified here. That most compounds are both rapidly taken up and released by cells suggests that the plateau phase represents an equilibrium state during which time any compound that is released is subject to re-entry as long as it remains in its pro-drug state. The period over which this equilibrium is maintained is dependent both on the total amount of available extracellular prodrug and its rate of conversion to JY-3-094 within the cell.

## MATERIALS AND METHODS

### Chemical syntheses

Analogs of 10074-G5 shown in Supplementary Fig. S1 and hereafter referred to as “Group A” compounds were prepared as described previously [28], or in Supplementary Materials and Methods. Pro-drugs derived from JY-3-094, hereafter referred to as ”Group B” compounds (Supplementary Fig. S2), were prepared as shown in Supplementary Fig. S3. Briefly, commercially available 4-chloro-7-nitrobenzofurazan (compound 1: Supplementary Fig. S3) was reacted with 4-aminobenzoic acid to generate JY-3-094. The ester pro-drugs were then obtained either by direct alkylation of the *para-*carboxylic acid of JY-3-094 with the appropriate alkylating agent, or through a condensation reaction mediated by *O*-(benzotriazol-1-yl)-*N,N,N′,N′*-tetramethyluronium hexafluorophosphate (HBTU). The structures of the six Group B compounds (3jc-91-1, 3jc-91-2, 3jc-91-3, 3jc-91-5, 3jc-91-7 and SF-4-017) are given in full in Supplementary Fig. S2.

### Myc-Max electrophoretic mobility shift assays (EMSAs)

EMSAs were performed essentially as described utilizing bacterially-expressed recombinant proteins purified nearly to homogeneity[21, 23]. Experiments with Max homodimers utilized the His_6_-tagged 160 amino acid isoform of the protein, termed Max(L) [43], which homodimerizes and binds DNA well [23, 44]. For studies with Myc-Max heterodimers, we used the His_6_-tagged 151 residue isoform of Max [Max(S)], which binds DNA well as a heterodimer with Myc but poorly as a homodimer [23, 44, 45]. Recombinant Myc protein, expressed in the pET151 vector, consisted of the His_6_-TEV protease substrate-tagged 85 amino acid bHLH-ZIP domain [23]. Each protein was purified by Ni-agarose affinity chromatography as previously described [23, 44, 45]. The His_6_-TEV tag was removed from the Myc protein by TEV protease cleavage followed by re-purification using Ni-agarose affinity chromatography to remove the His_6_-TEV protease and the cleaved His_6_-TEV tag as previously described [23]. Binding assays were performed with 30 nM of each protein and 30 nM of a 6-carboxy-2',4,4',5',7,7'–hexachlorofluorescein (HEX)-tagged double-stranded oligonucleotide containing a consensus Myc binding site (IDT, Coralville, IA) [23, 46].

### Cell lines and growth inhibition assays

Human embryonal kidney (HEK) cells were maintained in Dulbecco's-modified Eagle's minimal essential medium (D-MEM) supplemented with 10% fetal bovine serum (FBS), glutamine and penicillin/streptomycin as previously described [47]. Human breast carcinoma MDA-MB231 cells, obtained from the American Type Culture collection (Manassas, VA), were cultured in Dulbecco's modified Eagle medium (DMEM) supplemented with 10% FBS. Daudi Burkitt lymphoma and HL60 promyelocytic leukemia cells [23] were maintained in RPMI medium supplemented as described above for D-MEM. For growth inhibition studies, 3 × 10^3^ cells were seeded in 96 well plates in the presence of serial dilutions of each compound. A total of 10 dilutions, ranging from 0.1 to 50 μM were performed for each compound. MTT assays [23] were used to quantify cell number 3-4 d later and compared to wells exposed to DMSO vehicle only. Each point was assayed in quadruplicate with the results being presented as the mean +/- 1 standard error.

### Co-immunoprecipitation (Co-IP) assays

Co-IP assays were performed essentially as described previously [23]. Briefly, 5 × 10^6^ HL60 cells (>90% viable) in log-phase growth were treated in suspension for 4-6 hr with the stated concentration of Myc inhibitor. As a negative control, samples exposed to DMSO vehicle only were included and processed in parallel. Treated cells were collected by centrifugation, washed twice in ice-cold PBS and lysed in IP Buffer [23]. 300 μg of cleared lysate was then precipitated with a 1:200 dilution of anti-Max antibody [23, 44] followed by subsequent precipitation with protein G-Sepharose using conditions recommended by the supplier (Santa Cruz Biotechnology, Inc. Santa Cruz, CA). The precipitate was washed three times in IP buffer, boiled in running buffer and resolved by 10% SDS-PAGE. After electrotransfer of the proteins to a PVDF membrane (Millipore Corp. Billercica, MA), the blot was probed with a 1:1000 dilution of anti-Myc monoclonal antibody (9E10, Santa Cruz Biotechnology, Santa Cruz, CA). Blots were developed using a Pierce ECL Plus enhanced chemiluminescence kit according to the directions of the supplier (Thermo-Fisher, Pittsburgh, PA).

### Live cell confocal imaging

6 × 10^5^ human embryonal kidney cells (HEK293) were plated in glass bottom 35 mm dishes (MatTek Co., Ashland, MA) and allowed to attach overnight at which point they were generally 60-80% confluent and in log-phase growth. Myc inhibitors were then added directly to the medium to the final indicated concentrations. Throughout the experiment, cells were maintained under a controlled environmental enclosure at 37C and in a 5% CO_2_ atmosphere. All images were taken with a LSM710 laser scanning confocal microscope (Carl Zeiss, Munich, Germany). The following settings were used for all Myc inhibitors: objective: Plan-Apochromat 20x/0.8 M27; Laser: Excitation 488 nm at 2%, Laser Pinhole 34 mm, pixel dwell 6.4 mm; Detection filter: 490-735, Digital Gain 1.0, Digital Offset 0.00, Master gain 740 (for cmpd) 276 (for DIC); Image size: 1024×1024; Z stack scan total: 20 μm for 12 stacks. Just prior to the addition of each compound, images were obtained from which backgrounds were subtracted from the signals obtained following compound addition. Images were obtained at least every 5 min. Image analysis and quantification were performed with histogram and mean of ROI functions with ZEN 2009 software.

### Preparation of samples for liquid chromatography/mass spectrometry (LC-MS/MS)

10074-G5 (Santa Cruz Biotechnology) was used as an internal standard. LC/MS grade water, acetonitrile (CH_3_CN), and formic acid were from Thermo-Fisher Scientific. HL60 cells were grown in suspension to approximately 10^6^ cells/ml and MDA-MB231 cells were grown in monolayer cultures to 70–80% confluency in 6 well plates. Fresh culture medium containing 10 μM of the indicated compounds was then added for 72 hours. Cells were harvested in ice-cold phosphate-buffered saline (PBS) and re-suspended in RIPA buffer (Sigma-Aldrich, St. Louis, MO) containing 1% Na_3_PO_4_, 0.5% Na-deoxycholate and 0.1% SDS, supplemented with protease inhibitor cocktail (Sigma-Aldrich). After a 10 min incubation on ice, the crude cell extracts were quick-frozen in liquid nitrogen and stored at –80°C.

### LC-MS/MS

10 μL of internal standard (10 μM 10074-G5) was added to each 100 μL of cell lysate and mixed for 30 sec. 500 μL of acetonitrile (CH_3_CN) was then added and immediately mixed for an additional 30 sec. After centrifugation at 10,000 × g for 5 min, 500 μl of supernatant was transferred to a new tube and evaporated to dryness under nitrogen at 30°C. The residue was reconstituted with 100 μL of water/ CH_3_CN (1:1 v/v) plus 0.1% formic acid.

LC/MS analysis was performed on a TSQ Quantum Ultra Triple Stage Quadrupole Mass Spectrometer coupled to an Ultimate 3000 RS Liquid Chromatogram system (Thermo Scientific, San Jose, CA). The LC separation was performed on an Acclaim 120 C18 column (2.1 × 50mm, 5 μm) (Thermo Scientific) operated at 30°C. Solvents A and B consisted of 0.1% formic acid in water and CH_3_CN, respectively. The gradient program was as follows: 0.0-0.25 min, 25% B; 0.25-1.25 min, gradient to 65% B; 1.25-2.5 min, gradient to 90% B; 2.5-4.25 min, 90% B; 4.25-4.75 min, gradient to 25% B; 4.75-6.0 min, 25% B. The flow rate was 0.4 mL/min during all separation steps and injection volume was 10 μL. Detection was performed in the negative-ion mode and the electrospray ionization (ESI) source parameters were as follows: spray voltage, 2750; capillary temperature, 320; sheath gas pressure, 40; ion sweep gas pressure, 5; capillary offset, -35; tube lens offset, 100. Selected reaction monitoring (SRM) was used for mass detection with the following transitions: 3JC91-1 (*m/z* 313.1 → 266.3), 3JC91-2 (*m/z* 389.1 → 342.3), 3JC91-3 (*m/z* 327.1 → 280.3), 3JC91-5 (*m/z* 371.1 → 324.3), 3JC91-7 (*m/z* 381.1 → 334.3), SF-4-017 (*m/z* 375.1 → 328.3), JY-3-094 (*m/z* 299.1 → 209.3), and 10074-G5 (*m/z* 331.1 → 284.3). Data collection and analysis were performed by Xcalibur V 2.1 (Thermo Scientific).

### LC-MS/MS Method Validation

Calibration standards were generated by adding known amounts of standards (range 0.05 μM to 25 μM) into neat solution (Water/CH_3_CN 1:1 with 0.1% formic acid) and blank cell lysates. The QC samples in the same concentration as the calibration standards were prepared separately. Calibration curves were obtained by plotting the peak area ratio of the standard to the internal standard against the concentration and linear regression curves were calculated to check the linearity of the method. Limit of detection (LOD) and limit of quantitation (LOQ) were determined as signal-to-noise ratio of 3 and 10, respectively. Supplementary Fig. S4 shows calibration curves with LOD and LOQ for each Group B prodrug and metabolite.

## SUPPLEMENTARY VIDEO1 AND FIGURES




