# Erratum: Disruption of Myc-Max Heterodimerization with Improved Cell-Penetrating Analogs of the Small Molecule 10074-G5

**DOI:** 10.18632/oncotarget.26241

**Published:** 2018-10-12

**Authors:** Huabo Wang, Jay Chauhan, Angela Hu, Kelsey Pendleton, Jeremy L. Yap, Philip E. Sabato, Jace W. Jones, Mariarita Perri, Jianshi Yu, Erika Cione, Maureen A. Kane, Steven Fletcher, Edward V. Prochownik

**Affiliations:** ^1^ Section of Hematology/Oncology, Children’s Hospital of Pittsburgh of UPMC; ^2^ Department of Pharmaceutical Sciences, University of Maryland School of Pharmacy, Baltimore, MD, USA; ^3^ PharmD Program, University of Maryland School of Pharmacy, Baltimore, MD, USA; ^4^ Department of Pharmaco-Biology, University of Calabria, Rende (CS), Italy; ^5^ University of Maryland Marlene and Stewart Greenebaum Cancer Center, Baltimore, MD, USA; ^6^ Department of Microbiology and Molecular Genetics, The University of Pittsburgh School of Medicine; ^7^ The University of Pittsburgh Cancer Institute, Pittsburgh, PA

**This article has been corrected:** During production, the ending page number for this article was listed incorrectly. The page count has now been adjusted to show the proper pagination.

Original article: Oncotarget. 2013; 4:936-947. https://doi.org/10.18632/oncotarget.1108

