# Supplementary material for: Disruption of Myc-Max Heterodimerization with Improved Cell-Penetrating Analogs of the Small Molecule 10074-G5
Source: Oncotarget. 2013 Jun 22;4(6):936–47. doi: 10.18632/oncotarget.1108 (PMC3757250; doi:10.18632/oncotarget.1108)
Supplement: Supplementary file 2 [file oncotarget-04-936-s002.pdf]

# Disruption of Myc-Max Heterodimerization with Improved Cell-Penetrating Analogs of the Small Molecule 10074-G5 - Wang et al

## Group A Compounds

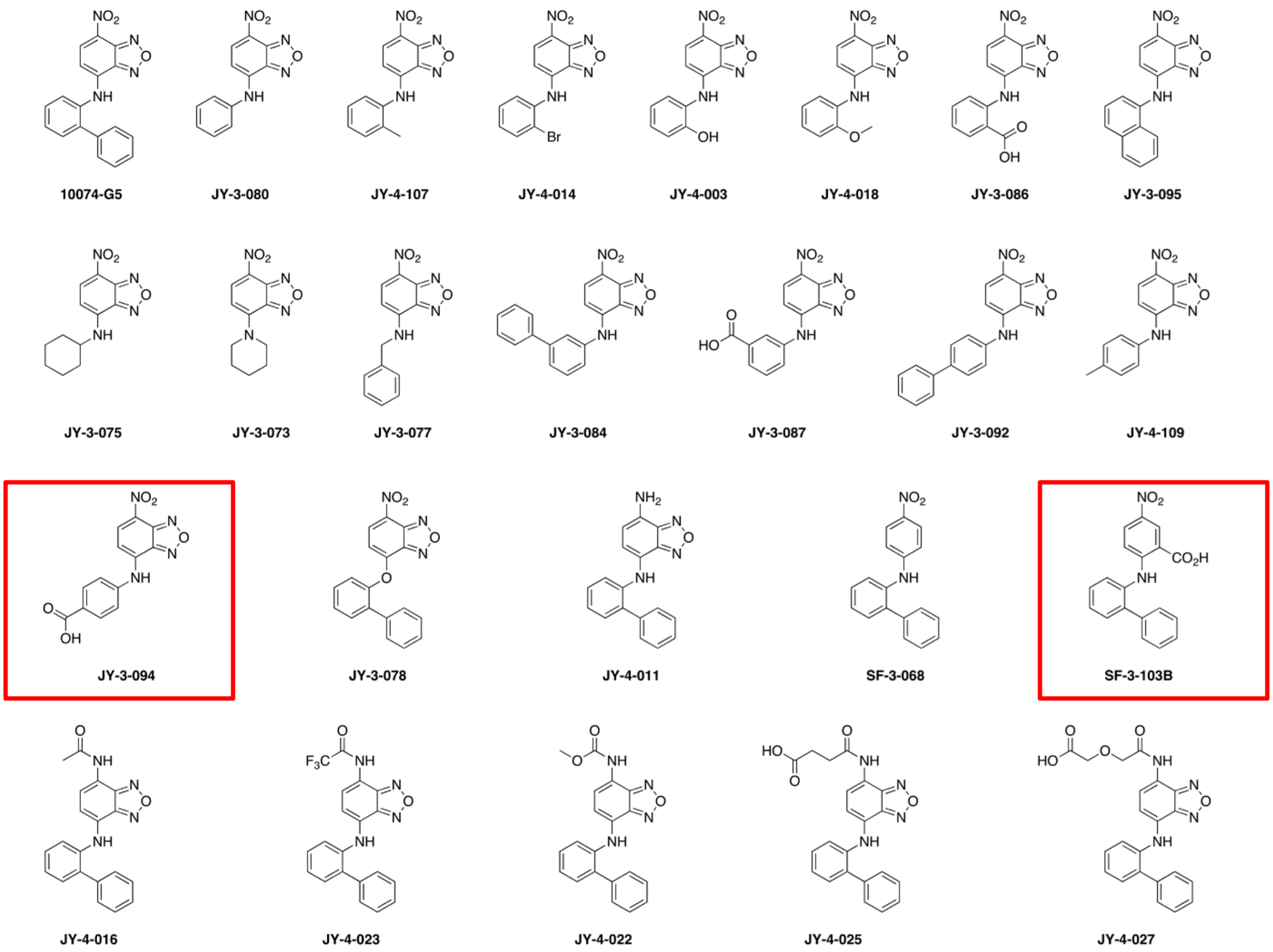


**Supplementary Figure S1: Group A compounds.** The structure of the previously described starting compound, 10074-G5 [21] is shown at the top left. Each 10074-G5 analog was tested at 50-100 µM in an EMSA-based assay employing purified, recombinant Myc and Max(S) proteins and a HEX-labeled E-box-containing oligonucleotide as previously described [23]. The two active analogs identified in this screen, JY-3-094 (28) and SF-3-103B, are boxed.

## Group B Compounds

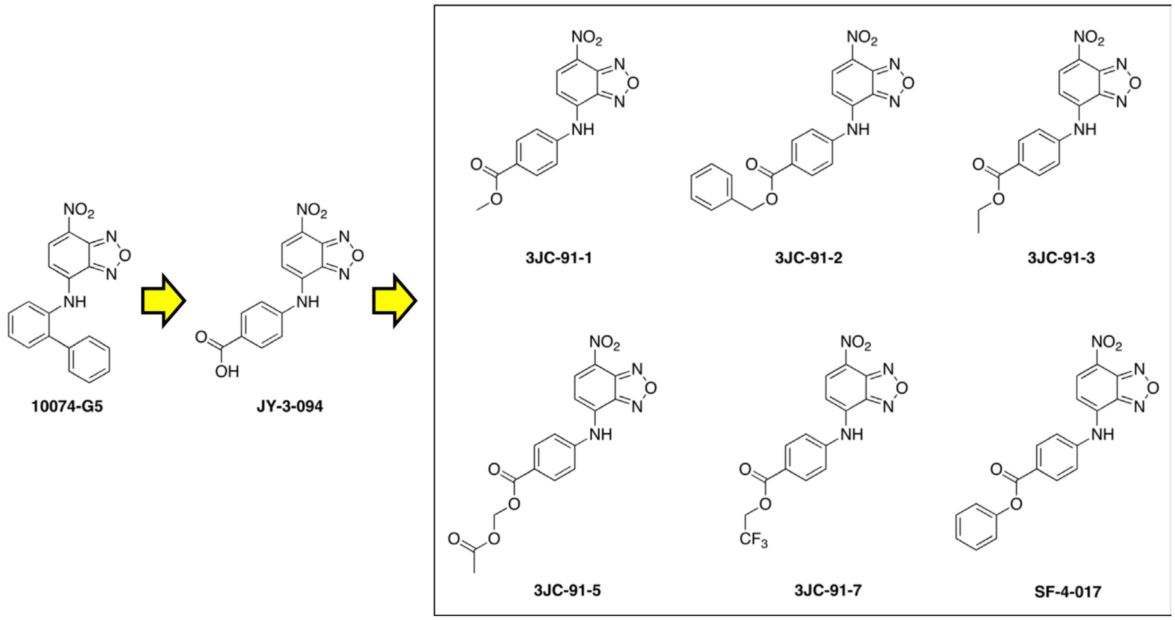


**Supplementary Figure S2: Group B compounds.** The carboxyl moiety of JY-3-094 was esterified with six different groups as outlined in Supplementary Fig. S3.

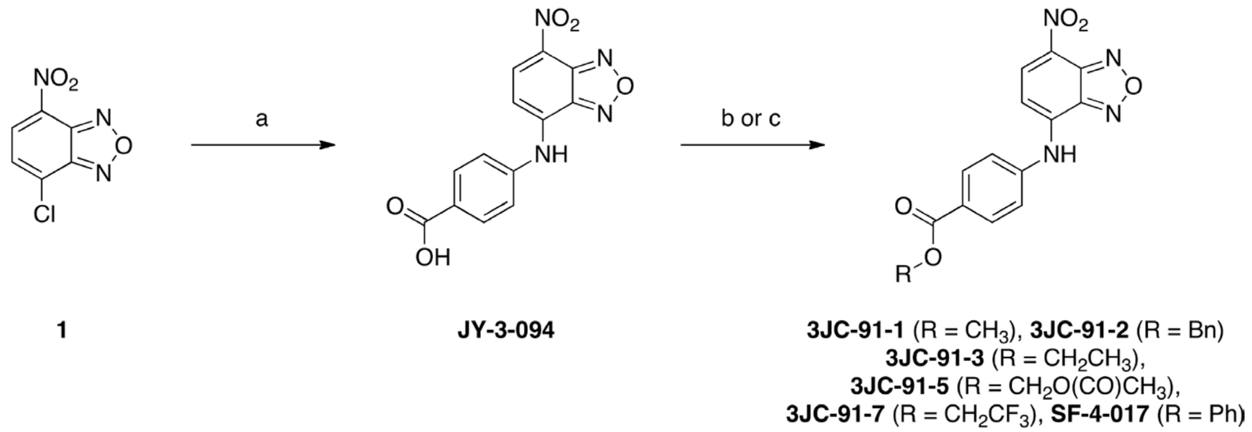


**Supplementary Figure S3: Derivation of Group B compounds from JY-3-094.** 4-chloro-7-nitrobenzofurazan (1). a) 4-aminobenzoic acid, *N*,*N*-diisopropylethylamine, $CH_3CN$, rt, 16 h, 15%; b) RBr or RI, $K_2CO_3$, DMF, rt, 6 h, 64 – 82%; c) PhOH, HBTU, *N*,*N*-diisopropylethylamine, DMF, rt, 16 h, 68%.

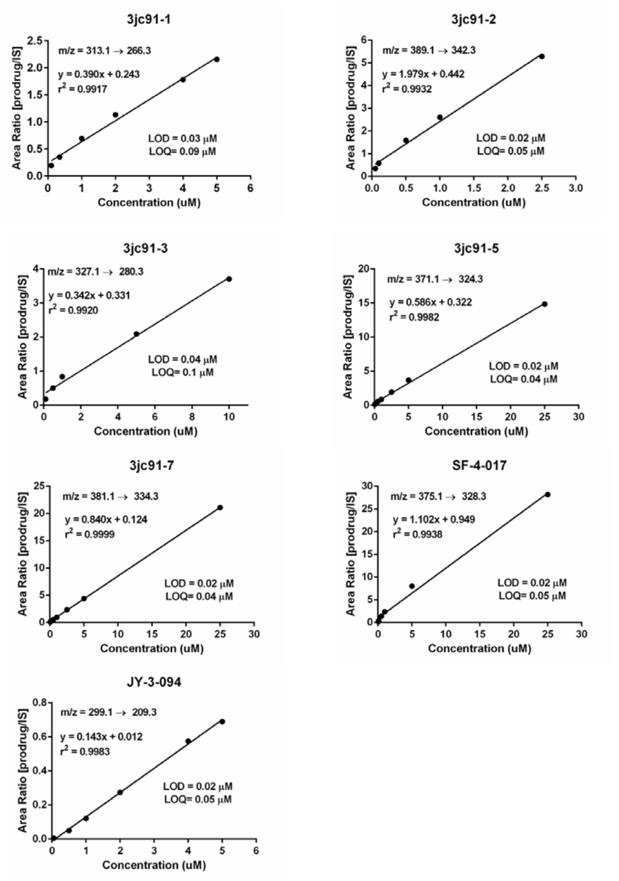


**Supplementary Figure S4: Calibration curves for each prodrug compound and the active metabolite (JY-3-094).** Equation of best fit, $r^2$, LOD (s/n ≥ 3) and LOQ (s/n ≥ 10) are listed for each compound.

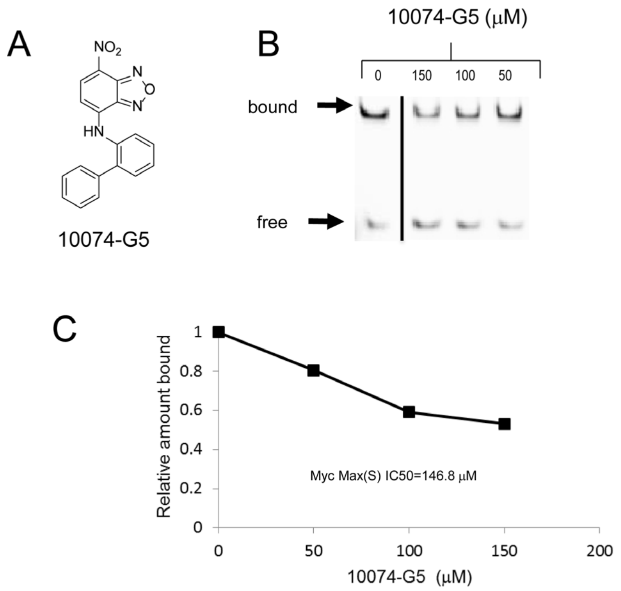


**Supplementary Figure S5: EMSA binding of parental compound 10074-G5 [21].** A, Structure of 10074-G5 (see also Suppl. Fig. S1). B, Typical EMSA results obtained with Myc-Max(S) heterodimers. Recombinant $His_6$-c-$Myc_{353\text{-}439}$ and full-length $His_6$-Max(S) were purified to homogeneity from *E. coli* and used at a final concentration of 30 nmol/L each in the presence of the indicated concentration of 10074-G5. A HEX$^{TM}$-tagged 22 bp E-box-containing dsDNA oligonucleotide was used at 30 nmol/L concentration in all reactions [23]. C, Quantitative analysis of EMSA. Similar concentrations of 10074-G5 failed to have any effect on DNA binding by Max(L) (not shown).

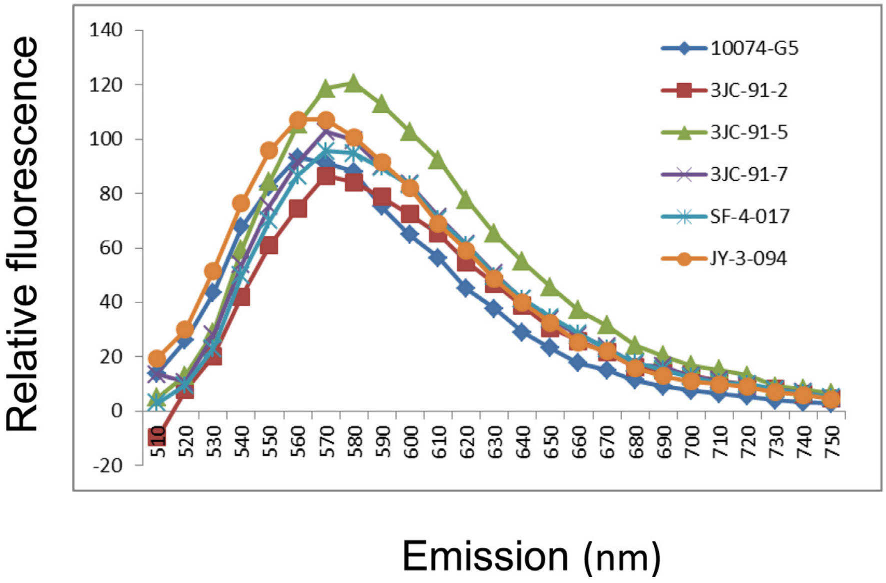


**Supplementary Figure S6: Excitation/Emission spectra for 10074-G5, JY-3-094 and Group B compounds.** Spectra were obtained on 500 μM solutions of each of the indicated compounds in DMSO (Excitation=470 nm).

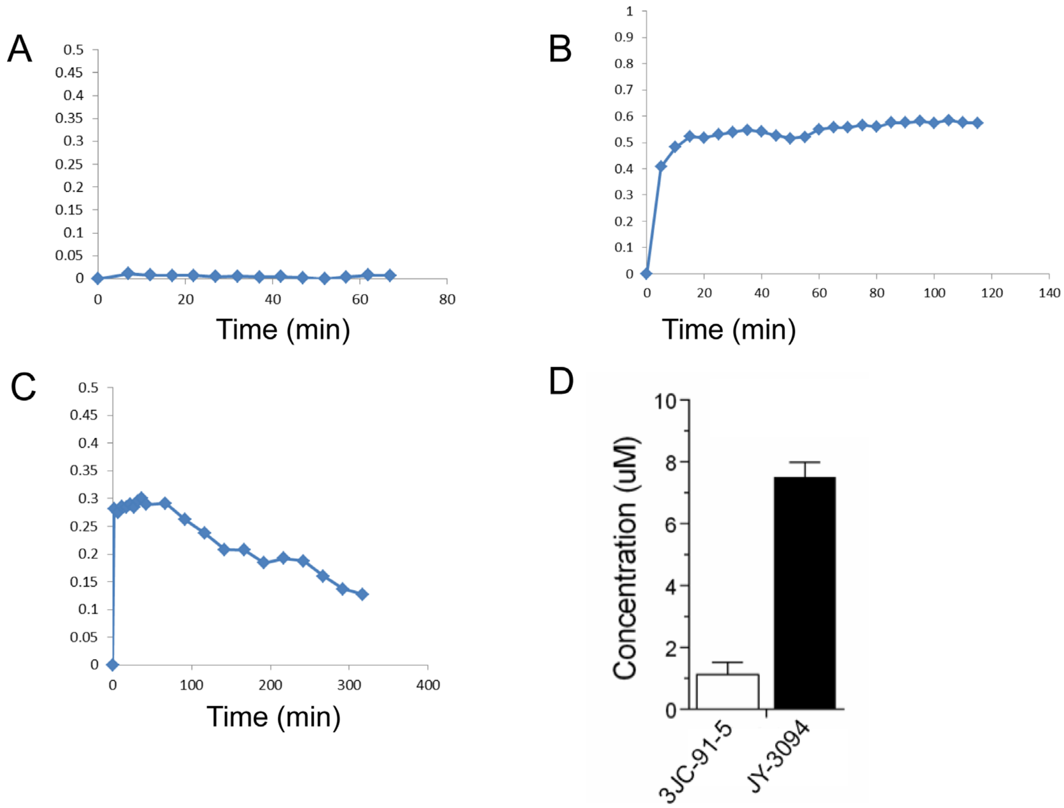


**Supplementary Figure S7: 3JC-91-5 is highly susceptible to esterases.** A, 3JC-91-5 is rapidly depleted from the extracellular compartment. HEK cells were exposed to 10 µM 3JC-91-5 for 3 hr. in medium + 10% serum as described in the legend to Fig. 3. A pattern of rapid compound uptake and subsequent loss of fluorescence was observed within this time, thus confirming the results of Fig. 3. The medium was removed and added to a fresh cell monolayer. Note that no significant uptake was seen under these conditions. B, 3JC-91-2 uptake in the presence of serum-containing medium was allowed to proceed as described in (A). The medium was then removed and added to fresh cells and uptake was monitored. C, Uptake of 3JC-91-5 in the absence of serum. A HEK cell monolayer was exposed to 10 µM 3JC-91-5 under conditions identical to those described above except that serum-free medium was used. Note the previously observed rapid uptake and subsequent decline of fluorescence indicating conversion to JY-3-094 by intracellular esterases. Also note that, in the absence of serum, the gradual loss of the 3JC-91-5 fluorescence occurs more slowly. D, 3JC-91-5 is converted to JY-3-094 in the absence of cells. 10 µM 3JC-91-5 in medium containing 10% FBS was incubated at 37C for 6 hr. The relative amounts of 3JC-91-5 and JY-3-094 were then determined by MS. Note that >80% of the former compound was converted to the latter over the course of the experiment

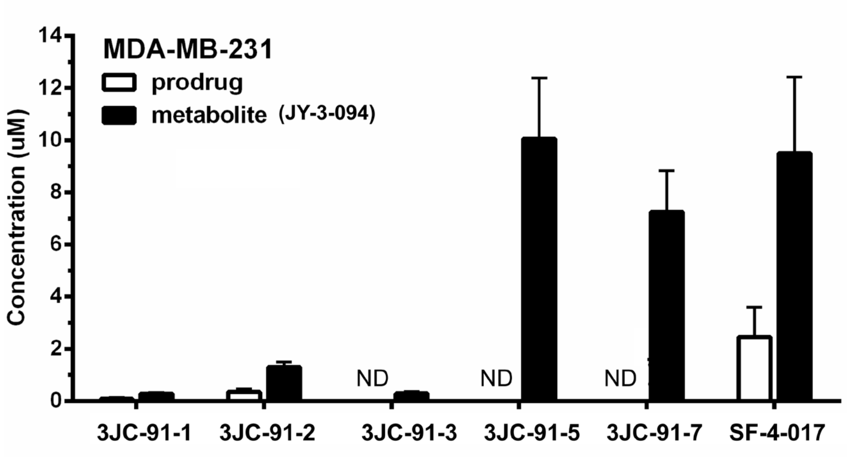


**Supplementary Figure S8: LC-MS/MS quantification of intracellular prodrug and JY-3-094 in MDA-MB231 cells.** Cells were cultured in the presence of 10 µM of each prodrug for 72 h and then intracellular prodrug and metabolite (JY-3-094) levels were quantified by LC-MS/MS. All data are mean ± standard deviation with N=3 for each prodrug treatment. ND.=not detectable.

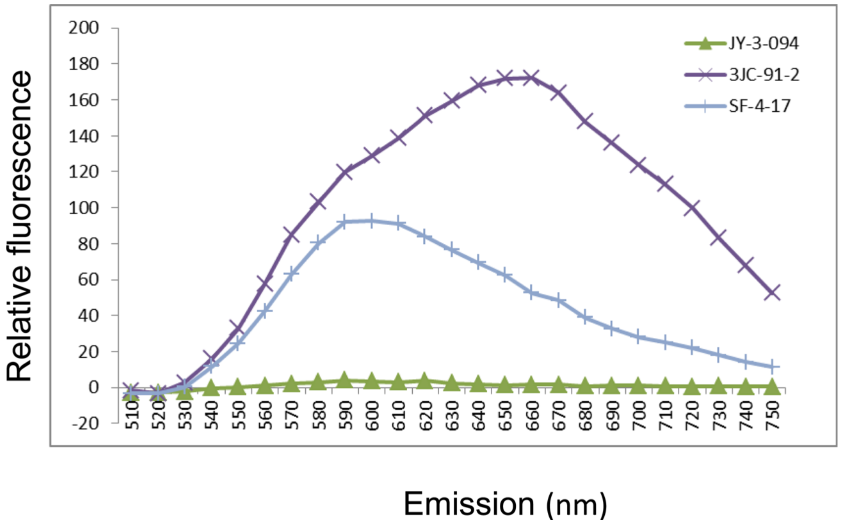


**Supplementary Figure S9: Loss of fluorescence of JY-3-094 in aqueous solution.** Spectrophoto-metric profiles on each of the indicated compounds (50 µM each in PBS) were generated as described for Supplementary Fig. S6.

# MATERIALS AND METHODS

## General Procedure 1

To a solution of JY-3-094 (28) (40 mg, 0.13 mmol) and $K_2CO_3$ (74 mg, 0.53 mmol) in DMF (2 mL) was added the corresponding alkyl halide RBr or RI (2 eq.) at room temperature. The reaction mixture was stirred for 6 h and partitioned between EtOAc and sat. $NH_4Cl$. The organic layer was washed with $H_2O$ (x 3), sat. NaCl, dried over $Na_2SO_4$ and reduced *in vacuo*. The crude residue was purified by column chromatography ($SiO_2$, Hexane/EtOAc) to provide the target compounds.

*Methyl 4-((7-nitrobenzo[c][1,2,5]oxadiazol-4-yl)amino)benzoate* (3JC-91-1). Following general procedure 1 with methyl iodide gave the title compound as a red solid (28 mg, 68 %). $\delta_H$ (DMSO-$d_6$, 400 MHz) 11.14 (br s, 1 H, NH), 8.76 (d, $J$ = 8.8, 1 H, Ar), 8.04 (d, $J$ = 7.6, 1 H, Ar), 7.62 (d, $J$ = 7.6, 1 H, Ar), 7.01 (d, $J$ = 8.8, 1 H, Ar), 3.86 (s, 3 H, $CH_3$); $\delta_C$ (DMSO-$d_6$, 100 MHz) 166.0, 145.8, 144.5, 143.1, 141.0, 137.6, 131.1, 126.5, 125.1, 122.8, 104.1, 52.6; *m/z* (APCI +ve) 315 $[M+H]^+$.

*Benzyl 4-((7-nitrobenzo[c][1,2,5]oxadiazol-4-yl)amino)benzoate* (3JC-91-2). Following general procedure 1 with benzyl bromide gave the title compound as a red solid (41 mg, 82 %). $\delta_H$ (DMSO- $d_6$, 400 MHz) 8.49 (d, $J$ = 8.8, 1 H, Ar), 8.21 (d, $J$ = 8.8, 2 H, Ar), 7.95 (br s, 1 H, NH), 7.51-7.29 (m, 7 H, Ar), 6.94 (d, $J$ = 8.8, 1 H, Ar), 5.40 (s, 2 H, $CH_2$); $\delta_C$ (DMSO-$d_6$, 100 MHz) 165.3, 145.0, 141.2, 139.3, 135.7, 135.4, 131.8, 128.7, 128.4, 128.3, 127.9, 121.7, 102.3, 67.0; *m/z* (APCI +ve) 391 $[M+H]^+$.

*Ethyl 4-((7-nitrobenzo[c][1,2,5]oxadiazol-4-yl)amino)benzoate* (3JC-91-3). Following general procedure 1 with ethyl bromide gave the title compound as a red solid (35 mg, 81 %). $\delta_H$ (DMSO-$d_6$, 400 MHz) 11.16 (br s, 1 H, NH), 8.57 (d, $J$ = 8.8, 1 H, Ar), 8.05 (d, $J$ = 7.6, 2 H, Ar), 7.63 (d, $J$ = 7.6, 2 H, Ar), 7.01 (d, $J$ = 8.8, 1 H, Ar), 4.33 (q, $J$ = 7.2, 2 H, $CH_2$), 1.33 (t, $J$ = 7.2, 3 H, $CH_3$); $\delta_C$ (DMSO- $d_6$, 100 MHz) 165.1, 154.4, 144.2, 142.7, 140.8, 137.2, 130.7, 126.4, 124.6, 122.4, 103.7, 60.8, 14.2; *m/z* (APCI +ve) 329 $[M+H]^+$.

*Acetoxymethyl 4-((7-nitrobenzo[c][1,2,5]oxadiazol-4-yl)amino)benzoate* (3JC-91-5). Following general procedure 1 with bromomethyl acetate gave the title compound as a red solid (31 mg, 64 %). $\delta_H$ (DMSO-$d_6$, 400 MHz) 11.17 (br s, 1 H, NH), 8.58 (d, $J$ = 8.8, 1 H, Ar), 8.06 (d, $J$ = 8.0, 2 H, Ar), 7.65 (d, $J$ = 8.0, 2 H, Ar), 7.06 (d, $J$ = 8.8, 1 H, Ar), 5.94 (s, 2 H, $CH_2$), 2.12 (s, 3 H, $CH_3$); $\delta_C$ (DMSO- $d_6$, 100 MHz) 169.9, 164.3, 145.9, 144.5, 143.9, 140.8, 137.6, 131.6, 125.4, 125.2, 122.7, 104.5, 80.1, 21.0; *m/z* (APCI +ve) 373 $[M+H]^+$.

*Trifluoroethyl 4-((7-nitrobenzo[c][1,2,5]oxadiazol-4-yl)amino)benzoate* (3JC-91-7). Following general procedure 1 with 2-bromo-1,1,1-trifluoroethane gave the title compound as a red solid (38 mg, 76 %). $\delta_H$ ($CDCl_3$, 400 MHz) 8.48 (d, $J$ = 8.8, 1 H, Ar), 8.18 (d, $J$ = 8.4, 2 H, Ar), 7.50 (d, $J$ = 8.4, 2 H, Ar), 6.97 (d, $J$ = 8.4, 1 H, Ar), 4.71 (q, $J$ = 7.6, 1 H, Ar); $\delta_C$ ($CDCl_3$, 100 MHz) 163.8, 145.1, 143.7, 142.1, 138.8, 135.2, 132.2, 125.9, 121.6, 102.6, 61.4, 60.8; *m/z* (APCI +ve) 383 $[M+H]^+$.

*Phenyl 4-((7-nitrobenzo[c][1,2,5]oxadiazol-4-yl)amino)benzoate* (SF-4-017). To aryl acid (50 mg, 0.17 mmol) and HBTU (74 mg, mmol) in DMF (2 mL) was added DIPEA (58 mL, 0.33 mmol) at room temperature. After 1 h, phenol (24 mg, 0.25 mmol) was added and the reaction mixture was stirred for 20 h before being partitioned between EtOAc and sat. $NH_4Cl$. The organic layer was washed with $H_2O$ (x 3), sat. NaCl, dried over $NaSO_4$ and reduced *in vacuo*. The crude residue was purified by column chromatography ($SiO_2$, Hexane/EtOAc) to provide the title compound (28 mg, 68 %). $\delta_H$ (DMSO-$d_6$, 400 MHz) 11.21 (br s, 1 H, NH), 8.60 (d, $J$ = 8.8, 1 H, Ar), 8.22 (d, $J$ = 8.4, 2 H, Ar), 7.71 (d, $J$ = 8.4, 2 H, Ar), 7.49 (t, $J$ = 8.0, 2 H, Ar), 7.35-7.28 (m, 3 H, Ar), 7.08 (d, $J$ = 8.8, 1 H, Ar); $\delta_C$ (DMSO- $d_6$, 100 MHz) 164.3, 151.1, 145.9, 144.6, 143.9, 140.9, 137.6, 131.9, 130.0, 126.5, 125.6, 125.4, 122.7, 122.4, 104.5; *m/z* (APCI +ve) 377 $[M+H]^+$.

*Synthesis of 2-([1,1'-biphenyl]-2-ylamino)-5-nitrobenzoic acid* (SF-3-103B). To a solution of 2-fluoro-5-nitrobenzonitrile (690 mg, 4.13 mmol, 1 equiv) and 2-aminobiphenyl (700 mg, 4.13 mmol, 1 equiv) in anhydrous DMSO (20 mL) at room temperature was added potassium *tert*-butoxide (930 mg, 8.26 mmol, 2 equiv). After 16 h, the reaction mixture was diluted with water, and extracted into EtOAc (x3). The EtOAc extractions were combined, washed with water (x5), brine, dried ($Na_2SO4$), filtered and concentrated. The crude residue was dry-loaded onto silica gel and purified by flash column chromatography ($CH_2Cl_2$/Hex/EtOAc, 10:10:1) to afford 2-([1,1'-biphenyl]-2-ylamino)-5-nitrobenzonitrile as an orange solid (728 mg, 56%): $□_H$ ($CDCl_3$, 400 MHz) 8.31 (d, $J$ = 2.4, 1 H, Ar), 8.10 (dd, $J$ = 9.6, 2.4, 1 H, Ar), 7.50-7.24 (m, 9 H, Ar), 6.92 (d, $J$ = 9.6, 1 H, Ar), 6.79 (s, 1 H, NH); $□_C$ ($CDCl_3$, 100 MHz) 152.2, 138.5, 137.9, 137.5, 134.4, 131.6, 129.7, 129.5, 128.9, 128.8, 128.6, 128.2, 127.6, 125.4, 115.2, 112.5, 96.7;), which was subsequently hydrolyzed (1.17 mmol scale) with NaOH (140 mg, 3.52 mmol, 2 equiv) in a 1:1:1 mixture of dioxane/EtOH/$H_2O$ (12 mL) at reflux for 48 h. The reaction mixture was concentrated to dryness, and then partitioned between $Et_2O$ and $H_2O$ to remove neutral organics. The aqueous layer was acidified with 1M HCl, and then extracted with EtOAc (x3). The EtOAc extractions were combined, washed with brine, dried ($Na_2SO_4$), filtered and concentrated. The crude residue was dry-loaded onto silica gel and purified by flash column chromatography ($CH_2Cl_2$/MeOH/AcOH, 92:7:1) to deliver the title compound 2-([1,1'-biphenyl]-2-ylamino)-5-nitrobenzoic acid (SF-3-103B) as an orange solid (293 mg, 75%): $□_H$ ($CDCl_3$+MeOH-$d_4$, 400 MHz) 10.01 (s, 1 H, OH), 8.80 (d, $J$ = 2.4, 1 H, NH), 7.98 (dd, $J$ = 9.6, 2.4, 1 H, Ar), 7.43-7.17 (m, 11 H, Ar), 6.82 (d, $J$ = 9.6, 1 H, Ar); $□_C$ ($CDCl_3$, 100 MHz) 169.2, 153.2, 138.3, 138.2, 137.0, 135.4, 131.4, 129.5, 129.1, 128.8, 128.5, 128.4, 127.5, 126.9, 126.0, 113.0, 110.2.
